# Classification and clinical significance of immunogenic cell death-related genes in *Plasmodium falciparum* infection determined by integrated bioinformatics analysis and machine learning

**DOI:** 10.1186/s12936-024-04877-3

**Published:** 2024-02-15

**Authors:** Yan-hui Zhang, Li-hua Xie, Jian Li, Yan-wei Qi, Jia-jian Shi

**Affiliations:** 1 Key Laboratory of Gastrointestinal Cancer (Fujian Medical University), Ministry of Education, Fuzhou, China; 2 State Key Laboratory of Cellular Stress Biology, Innovation Center for Cell Signaling Network, School of Life Sciences, Xiamen University, Xiamen, Fujian China; 3 Department of Pathogenic Biology and Immunology, School of Basic Medical Sciences, Guangzhou Medical University, Guangzhou, China

**Keywords:** Immunogenic cell death (ICD), *Plasmodium falciparum*, Machine learning, *CD3E*, *FCGR1A*

## Abstract

**Background:**

Immunogenic cell death (ICD) is a type of regulated cell death that plays a crucial role in activating the immune system in response to various stressors, including cancer cells and pathogens. However, the involvement of ICD in the human immune response against malaria remains to be defined.

**Methods:**

In this study, data from *Plasmodium falciparum* infection cohorts, derived from cross-sectional studies, were analysed to identify ICD subtypes and their correlation with parasitaemia and immune responses. Using consensus clustering, ICD subtypes were identified, and their association with the immune landscape was assessed by employing ssGSEA. Differentially expressed genes (DEGs) analysis, functional enrichment, protein-protein interaction networks, and machine learning (least absolute shrinkage and selection operator (LASSO) regression and random forest) were used to identify ICD-associated hub genes linked with high parasitaemia. A nomogram visualizing these genes' correlation with parasitaemia levels was developed, and its performance was evaluated using receiver operating characteristic (ROC) curves.

**Results:**

In the *P. falciparum* infection cohort, two ICD-associated subtypes were identified, with subtype 1 showing better adaptive immune responses and lower parasitaemia compared to subtype 2. DEGs analysis revealed upregulation of proliferative signalling pathways, T-cell receptor signalling pathways and T-cell activation and differentiation in subtype 1, while subtype 2 exhibited elevated cytokine signalling and inflammatory responses. PPI network construction and machine learning identified *CD3E* and *FCGR1A* as candidate hub genes. A constructed nomogram integrating these genes demonstrated significant classification performance of high parasitaemia, which was evidenced by AUC values ranging from 0.695 to 0.737 in the training set and 0.911 to 0.933 and 0.759 to 0.849 in two validation sets, respectively. Additionally, significant correlations between the expressions of these genes and the clinical manifestation of *P. falciparum* infection were observed.

**Conclusion:**

This study reveals the existence of two ICD subtypes in the human immune response against *P. falciparum* infection. Two ICD-associated candidate hub genes were identified, and a nomogram was constructed for the classification of high parasitaemia. This study can deepen the understanding of the human immune response to *P. falciparum* infection and provide new targets for the prevention and control of malaria.

**Supplementary Information:**

The online version contains supplementary material available at 10.1186/s12936-024-04877-3.

## Background

Malaria continues to be one of the most serious infectious diseases worldwide. There were an estimated 247 million cases of malaria and 619,000 malaria deaths worldwide in 2021, with sub-Saharan Africa bearing the highest proportions of cases [1]. A preponderance of fatalities can be traced back to infections with *Plasmodium falciparum*. The absence of an effective malaria vaccine, combined with the emergence of drug-resistant strains, are the most significant barriers in efforts to eradicate malaria. The complex relationship between the host and the *Plasmodium* parasite is critical for the progression and outcome of the disease, and host immunity plays a significant role in controlling the infection.

Immunogenic cell death (ICD), a distinct form of regulated cell death, is characterized by the release of specific danger signals from dying cells to the immune system [2]. Immunogenic cell death has important implications for cancer research, infectious diseases, and autoimmunity, as it can be harnessed to enhance antitumour immune responses, develop strategies against pathogens, and understand autoimmune diseases [3, 4]. However, there is limited research on the involvement of ICD in the human immune response against malaria. Understanding whether and how ICD is induced by *Plasmodium* infection can help us gain a better understanding of malaria pathogenesis and expedite the identification of effective targets for malaria control and prevention. Hyperparasitaemia is considered a severity criterion for malaria [5] and has been associated with a poor outcome in severe malaria [6]. Thus, appreciating how the human immune landscape influences the parasitaemia of *P. falciparum* will help expedite the rational design of more effective malaria vaccines.

In this paper, to investigate the potential involvement of ICD in the host immune response to *P. falciparum* infection, two ICD-associated subtypes were identified in the *P. falciparum* infection cohort by consensus clustering. The ICD-associated subtypes were found to have a significant correlation with parasitaemia and the host immune response induced by malaria parasites. Then, differentially expressed genes (DEGs) in different ICD subtypes and the related signalling pathways were identified. The candidate hub genes associated with ICD were identified by protein‒protein interaction network construction and machine learning. Then, a nomogram to visually represent the correlation between these ICD-associated hub genes and parasitaemia levels was constructed and evaluated by ROC analysis of the initial training cohort and two external validation cohorts.

Furthermore, the expression levels of the hub genes associated with ICD were found to be significantly correlated with clinical manifestations in *P. falciparum* infection. This investigation provides crucial insights for a better understanding of host–parasite interactions and offers valuable information for developing strategies to effectively control malaria.

## Methods

### Datasets

The whole-blood gene expression profiles and corresponding clinical data from patients with *P. falciparum* infection were obtained from the Gene Expression Omnibus (GEO). These data sets originated from cross-sectional studies, providing a comprehensive insight into the genetic landscape of this infection. The dataset GSE34404 served as the training cohort, while GSE132050 and GSE52166 were utilized as the validation cohorts. The GSE34404 dataset encompasses a cohort of 94 children, all under 10 years of age (median age 3.7), undergoing the symptomatic phase of blood-stage *P. falciparum* infection in urban Cotonou and rural Zinvié. These cases of uncomplicated acute malaria were confirmed through rapid diagnostic tests and blood smear analysis. The control group, comprising 61 age-matched individuals from Cotonou, tested negative for malaria and did not have sickle-cell disease. The peripheral blood samples from the malaria cases were collected prior to treatment.

The transcriptome data of malaria cases and control group was used in this study [7]. The GSE132050 dataset derives from a Controlled Human Malaria Infection (CHMI) study conducted at the Centre for Clinical Vaccinology and Tropical Medicine, Oxford. This dataset involved 14 adult volunteers who received intravenous injections of 690 *P. falciparum*-infected erythrocytes.The parasitaemia was monitored by qPCR twice daily with additional assessments using thick blood smears. The transcriptome data of whole blood at the day of diagnosis was used for analysis in this study [8]. The GSE52166 dataset forms a subset of a comprehensive cohort study focused on naturally acquired malaria immunity, conducted in Kalifabougou, Mali. This region is characterized by intense and seasonally-concentrated *P. falciparum* malaria transmission, typically spanning from June to December. The RNA sequencing analysis within this dataset included participants who were sampled at the time of their initial PCR-confirmed *P. falciparum* infection. This analysis encompassed a total of 41 samples from individuals aged between 6 and 23 years, each accompanied by corresponding parasitaemia data. The parasitaemia in GSE52166 dataset was detected by microscopic examination of blood smears [9, 10]. For GSE34404 and GSE132050 microarray datasets, the matrix.txt files and the corresponding platform annotation files were downloaded from GEO. The Limma package (version 3.52.4) in R software (version 4.2.1) was applied to perform background correction, normalization and log2 conversion for the matrix data of each GEO dataset with default settings. The platform annotation file was used to convert the probes into gene symbols, and the mean expression level of multiple probes that corresponded to the same gene symbol was taken as the expression level of the corresponding gene. For GSE52166 RNA-Seq dataset, the counts data of each sample and the matrix.txt file were downloaded from the GEO. Initially, batch effect verification was conducted to identify and correct for any potential batch-related variations, utilizing the ‘ComBat’ function from the sva package in R. Then gene identifiers were resolved to their corresponding gene symbols using the comprehensive gene annotation resources available through the Ensembl genome browser, facilitated by the biomaRt package in R. The expression values from multiple reads or transcripts mapping to the same gene were aggregated into a single gene-level measure by calculating their sum, utilizing the aggregate function in R. Finally, the processed data underwent normalization with the Trimmed Mean of M-values (TMM) using the edgeR package in R. Then a log2 transformation was performed on the normalized read counts. For both GSE132050 and GSE52166 datasets, malaria parasitaemia below 10,000 parasites per microlitre of blood was considered low parasitaemia, while levels exceeding this threshold were classified as high parasitaemia [11]. This high/low parasitaemia cutoff is close to the threshold in training dataset GSE34404. A cumulative clinical score for each patient in GSE132050 was calculated by summing adverse events on the day of diagnosis [8].

### Consensus clustering and characterization of parasitaemia and immune landscape between the two ICD subtypes

Genes associated with immunogenic cell death (ICD) were identified through a comprehensive literature analysis, which has been previously summarized by Garg et al. [12]. The inclusion criteria for the studies they considered were: (1) discussion of the correlation between ICD and danger signaling pathways or immune processes. (2) preventive or therapeutic rodent immunization experiments, or experiments involving the co-culture of cancer cells with immune cells. (3) experimental interventions, such as the use of siRNA/shRNA for gene silencing, antibody blocking, or whole/tissue/circuit-specific knockout models in rodents. (4) association of specific processes or molecular entities with ICD, substantiated by appropriate controls (untreated, negative, or positive). From their extensive survey, Garg et al*.* identified 33 ICD-related parameters, which equate to 34 distinct genes when CD8A and CD8B are counted separately. These parameters encompass a wide range of immunological complexity and represent both emerging and classic immune processes [12]. In the present study, the unsupervised clustering ‘PAM’ method based on Euclidean and average linkages was applied to identify distinct molecular subtypes based on ICD gene expression. The choice of the optimal number of clusters for PAM clustering was determined based on the Proportion of Ambiguous Clustering (PAC) method. This method is utilized to identify the most suitable number of clusters (k) by quantifying the ambiguity of the clustering results. The PAC score is calculated for each potential cluster number, and the optimal number of clusters is identified as the one with the lowest PAC score. The ConsensusClusterPlus tool in R was utilized to execute this procedure and identify molecular subtypes linked to ICD in the GSE34404 cohort. The process was repeated 5000 times to ensure classification stability. Principal component analysis (PCA) was conducted to show the distribution of ICD subtypes. To assess the clinical significance of the ICD subtypes, the association between ICD subtype and malaria parasitaemia was evaluated. Additionally, the correlations between ICD subtypes and immune cell populations in whole blood was assessed. The enrichment of 28 immune signatures for each sample was quantifed using the single-sample gene set enrichment analysis (ssGSEA) method, as implemented in the GSVA R package. The subsets of genes representing specific immune cell types were identified from the ImmPort database (Additional file 2: Table S1). The ssGSEA scores (normalized enrichment score, NES) were compared between different ICD subtypes. Statistical analyses were performed using unpaired t tests with adjustments for multiple comparisons made using the Benjamini–Hochberg (BH) procedure. All statistical analyses were conducted using R software (version 4.2.1).

### Identification of differentially expressed genes (DEGs) and functional enrichment analysis

The DEGs between the two ICD subtypes were identified using the “limma” R package. In addition, |log2-fold change (FC)| > 0.5 and p value < 0.05 were set as the criteria for identifying DEGs. Functional enrichment analyses, including Gene Ontology (GO) enrichment and gene set enrichment analysis (GSEA), were conducted with the R package clusterProfiler in the Bioconductor platform. GO enrichment analyses were premised on pvalueCutoff = 0.05 and qvalueCutoff = 0.01 thresholds. PvalueCutoff = 0.05 was designated as the threshold for GSEA.

### Identification of key ICD genes and machine learning

A PPI network of the DEGs between different ICD subtypes was constructed through the Search Tool for the Retrieval of Interacting Genes (STRING) database. Then, Cytoscape software v 3.8.0 was used to construct the PPI network. The top 10 upregulated genes and top 10 downregulated genes in ICD subtype 1 compared with ICD subtype 2 were identified using the CytoHubba plugin with the Maximal Clique Centrality (MCC) topological algorithm. Then, two machine learning algorithms were applied to further filter candidate genes for high parasitaemia classsification. The “glmnet” [13] and “randomForest” R packages were applied to perform LASSO regression [14] and RF analysis [15]. The genes identified by both LASSO and RF were considered potential hub genes for high parasitaemia classification.

### Nomogram construction and receiver operating characteristic (ROC) evaluation

A nomogram is a tool commonly used in medical prediction that combines the results of regression models and individual characteristics to estimate the probability of clinical outcomes [16]. Based on candidate hub genes, the “rms” R package was adopted to construct the nomogram. “Points” indicates the score of each candidate hub gene, and “Total Points” indicates the sum of all the scores of the genes above [17]. ROC evaluation was used to evaluate the classification performance of candidate genes and the nomogram in both the discovery cohort and the validation cohort using the “pROC” R package. The area under the curve (AUC) and 95% confidence interval (CI) were calculated to quantify the classification performance of the nomogram.

## Results

### Two ICD-associated subtypes were identified by consensus clustering

**Fig. 1:**
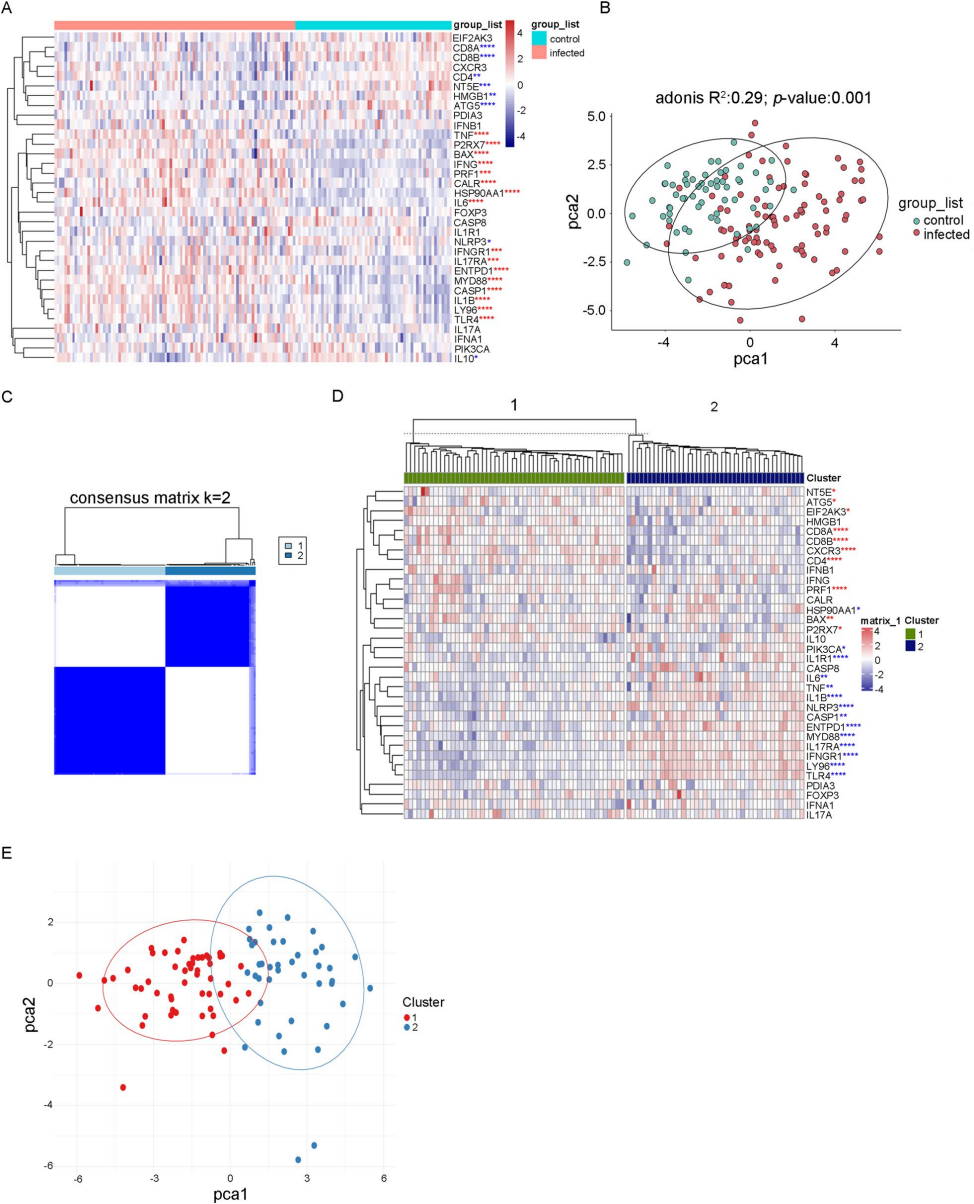
Two ICD-associated subtypes were identified in the *Plasmodium falciparum* infection cohort by consensus clustering. **A** Heatmap showing the expression patterns of ICD genes [12] in both uninfected control and malaria-infected patient samples in the GSE34404 dataset. The statistical analyses were performed employing an unpaired t-test, with adjustments for multiple comparisons made using the Benjamini–Hochberg (BH) procedure. * p < 0.05, ** p < 0.01, *** p < 0.001, **** p < 0.0001. Blue stars indicate lower expression, while red stars indicate higher expression in malaria-infected samples. **B** The PerMANOVA test based on the Euclidean distance measures showed a notable disparity of ICD genes expression between the control group and malaria-infected group in the GSE34404 dataset using principal component analysis (PCA) (adonis R^2^ 0.29; p value 0.001). **C** Consensus matrices of malaria-infected samples in the GSE34404 dataset for k = 2 using 5000 iterations of the unsupervised consensus clustering method (PAM) to ensure clustering stability. **D** Heatmap showing the expression of ICD genes in different subtypes in the GSE34404 dataset. Statistical analyses were conducted employing an unpaired t-test, with adjustments for multiple comparisons made using the Benjamini–Hochberg (BH) procedure. Blue stars and red stars indicate lower and higher expression in the cluster 1 subtype, respectively. **E** PCA of ICD subtypes in malaria-infected samples in the GSE34404 dataset

To investigate the potential involvement of ICD in the host immune response to *P. falciparum* infection, the expression patterns of ICD genes in both the uninfected control samples and malaria-infected samples in the GSE34404 dataset from a cross-sectional study were evaluated. In the malaria-infected samples, a subset of ICD genes, such as *TNF, P2RX7,BAX, IFNG, PRF1, CALR, HSP90AA1, IL6, IFNGR1, IL17RA, ENTPD1, MYD88, CASP1, IL1B, LY96* and *TLR4*, exhibited significant upregulation. Conversely, significant downregulation of numerous ICD genes, including *CD8A, CD8B, CD4, NT5E, HMGB1, ATG5, NLRP3* and *IL10,* was observed (Fig. 1A). GO enrichment of these genes indicated that malaria infection can induce cytokine production but can also suppress lymphocyte and mononuclear cell differentiation to a certain extent (Additional file 1: Fig. S1). Principal component analysis (PCA) was performed to show the difference in the expression of ICD genes between malaria-infected samples and uninfected control samples. Utilizing the non-parametric approach provided by the “vegan” package in R, PerMANOVA test based on the Euclidean distance measures revealed a notable disparity in ICD genes expression between these two groups, evidenced by an R^2^ value of 0.29 and a *p* value of 0.001 (Fig. 1B).

Subsequently, the ICD-associated subtypes amongst the malaria-infected samples were identified by employing consensus clustering techniques. Utilizing PAM clustering, two distinct clusters with divergent ICD gene expression patterns were identified within the malaria-infected samples from the GSE34404 dataset, including 52 cases in ICD-associated cluster 1 and 42 cases in ICD-associated cluster 2 (Fig. 1C). The transcriptomic profiles of ICD genes that were differentially expressed in the two clusters were delineated in a heatmap (Fig. 1D). Based on the results of the PCA, all malaria-infected samples could be approximately segregated into two distinct groups, further corroborating the existence of two remarkably divergent ICD subtypes (Fig. 1E). Therefore, the cluster 1 group was defined as ICD-associated subtype 1 and the cluster 2 group as ICD-associated subtype 2.

### The association of ICD subtype with parasitaemia and the immune cell profile in patients infected with *Plasmodium falciparum*

**Fig. 2:**
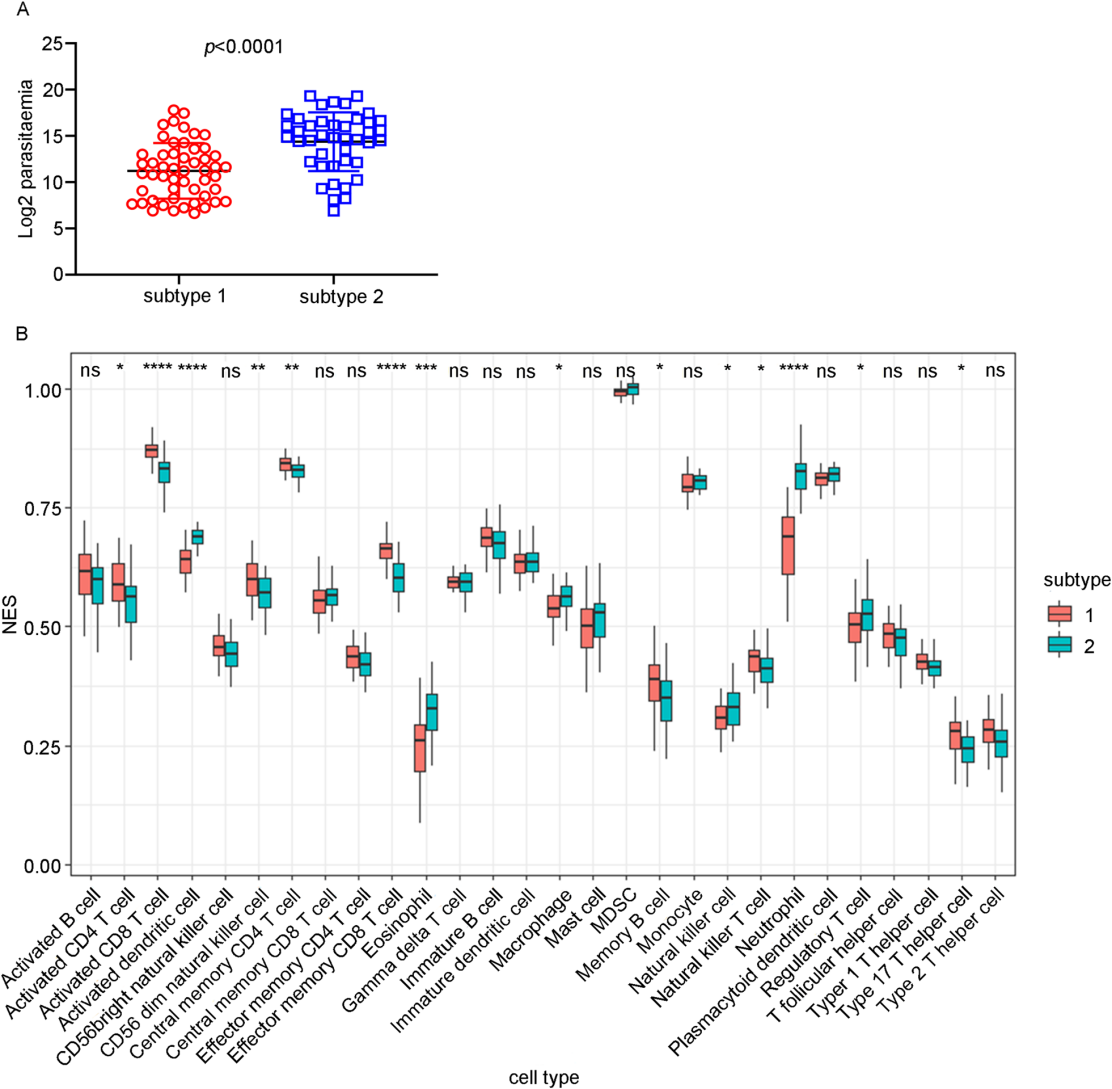
The ICD-association subtypes were correlated with parasitaemia and immune cell profile in *Plasmodium falciparum*-infected whole-blood samples. **A** Association of ICD subtypes with log2 parasitaemia in malaria-infected samples in the GSE34404 dataset. The statistical analysis was performed using an unpaired t test. **B** Immunophenotyping of different ICD subtypes in malaria-infected whole-blood samples in the GSE34404 dataset based on ssGSEA. The ssGSEA scores (enrichment level) were compared between different ICD subtypes. The statistical analyses were performed employing an unpaired t-test, with adjustments for multiple comparisons made using the Benjamini-Hochberg (BH) procedure. * p < 0.05, ** p < 0.01, *** p < 0.001, **** p < 0.0001

Then, to investigate whether ICD can affect the clinical phenotype of malaria infection, the relationship between the ICD subtypes and malaria parasitaemia was evaluated in the GSE34404 dataset. The results showed that the subtype 1 group had significantly lower parasitaemia than the subtype 2 group (Fig. 2A). This indicates that ICD may play a role in controlling parasite proliferation in the human host. To investigate whether the immune cell types in whole blood were affected by ICD, ssGSEA was used to assess and grade the enrichment of 28 immune signatures for each sample in both ICD subtypes. Compared to the subtype 2 group, the subtype 1 group had significantly more activated CD4 T cells, activated CD8 T cells, CD56-dim natural killer cells, central memory CD4 T cells, effector memory CD8 T cells, memory B cells, natural killer T cells and type 17 helper cells but significantly fewer activated dendritic cells, eosinophils, macrophages, natural killer cells, neutrophil and regulatory T cells (Fig. 2B). This result implied that subtype 1 is related to a better adaptive immune response, which may contribute to lower parasitaemia, compared with subtype 2.

### Differentially expressed genesecs (DEGs) and different signalling pathways were identified between the different ICD subtypes

**Fig. 3:**
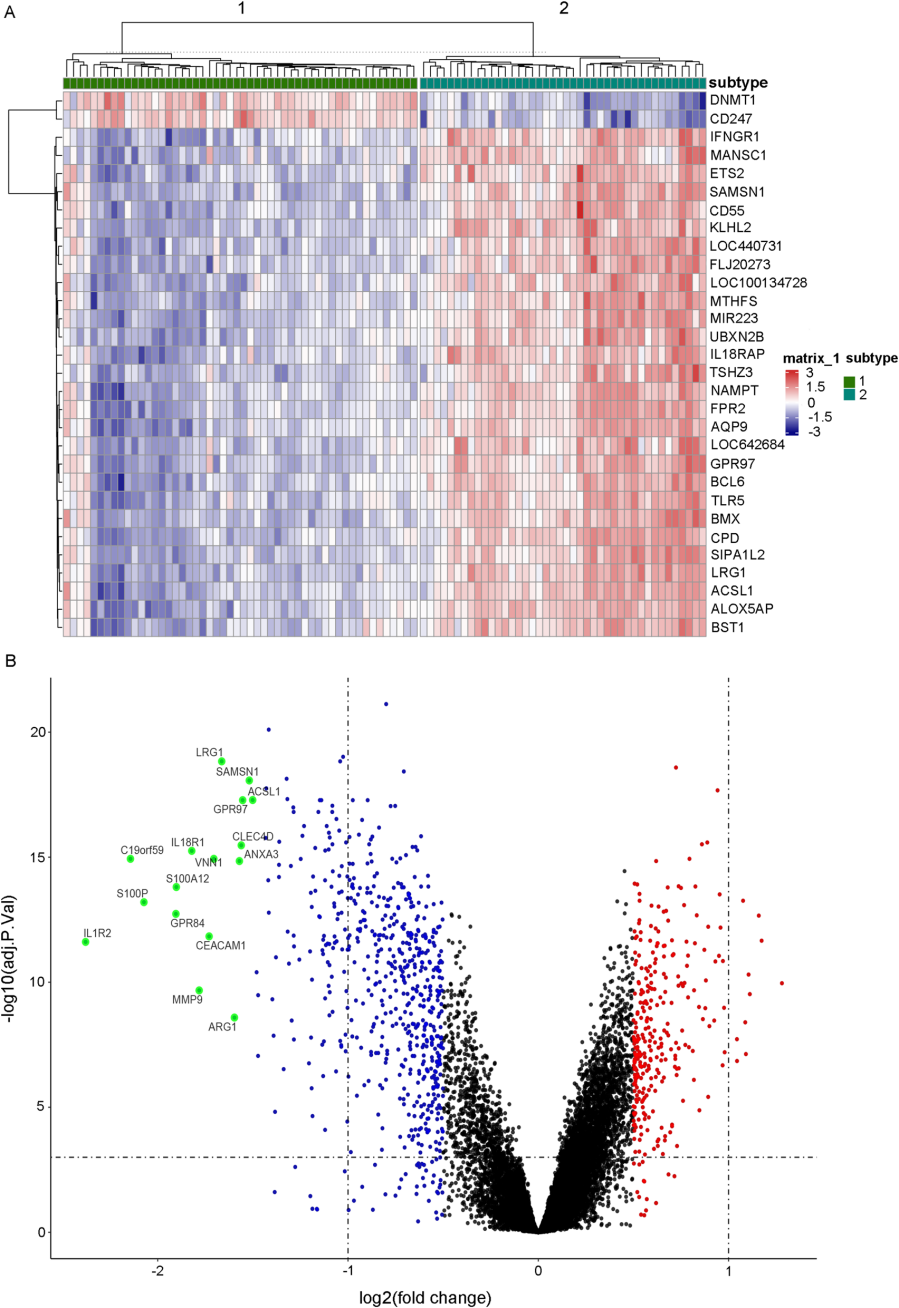
Differentially expressed genes (DEGs) were identified in different ICD subtypes. **A** Heatmap of the top 30 DEGs in the ICD subtype 1 group compared with the ICD subtype 2 group was constructed with a filtering threshold of a *p* value less than 0.05 and |log2-fold change| greater than 0.5 by using the limma algorithm. **B** Volcano plot presenting the distribution of DEGs between different ICD subtypes in infected samples in GSE34404; differentially expressed genes with |log2-fold change| greater than 1.5 are annotated

**Fig. 4:**
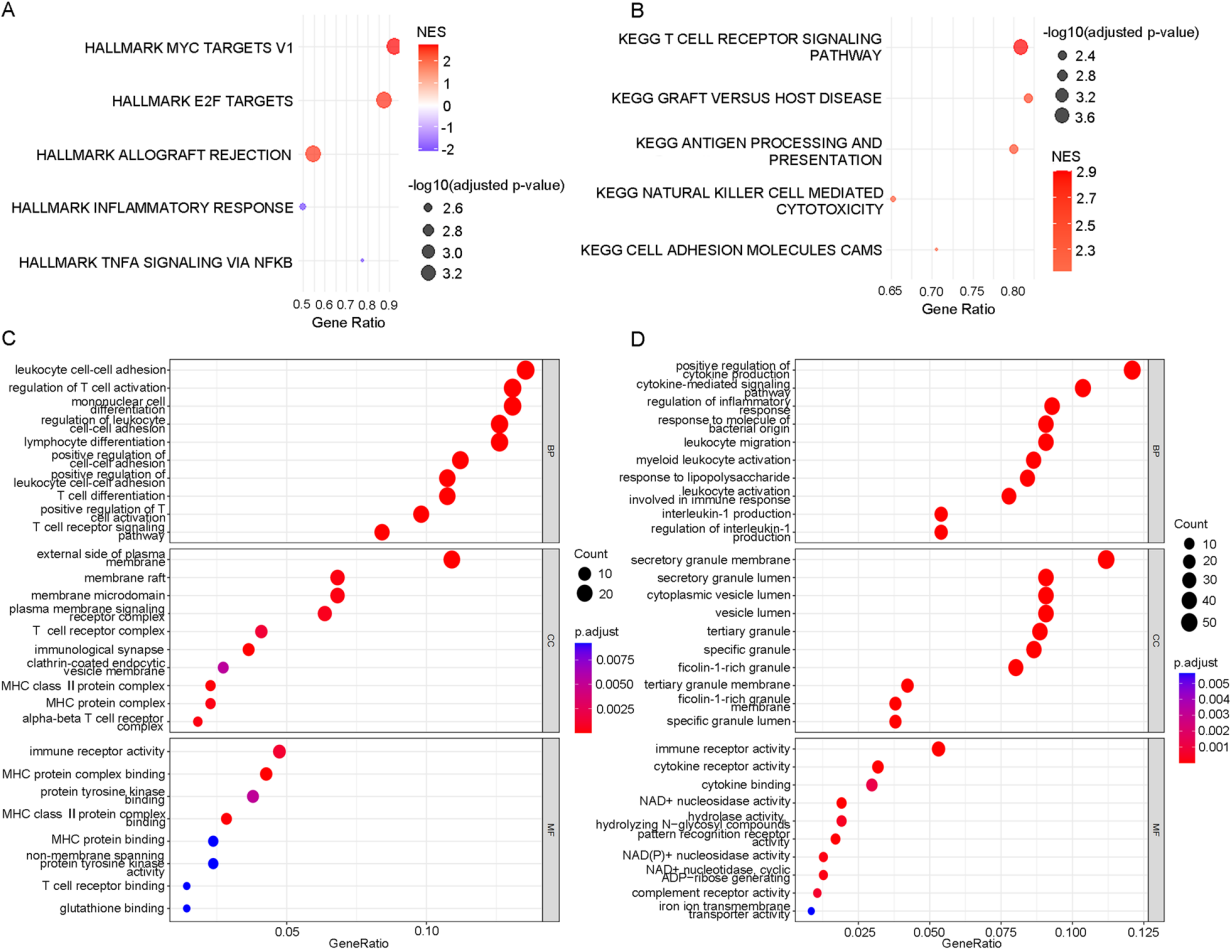
Functional enrichment of DEGs between the two ICD subtypes using the clusterProfiler package. **A** Hallmark GSEA of 910 DEGs between the ICD subtype 1 and ICD subtype 2 groups. **B** KEGG GSEA of DEGs between the ICD subtype 1 and ICD subtype 2 groups. For **A** and **B**, “Gene ratio”is the proportion of genes from a predefined gene set that appear in the ranked list of genes being analysed. The size of the dots represents -log10 of the P-adjusted values, and the colour of the dots represents the NES. **C** GO enrichment analysis of genes upregulated in the ICD subtype 1 group compared with the ICD subtype 2 group. **D** GO enrichment analysis of genes downregulated in the ICD subtype 1 group compared with the ICD subtype 2 group. For **C** and **D**, “Gene ratio” is the percentage of total DEGs annotated with the given GO term. The size of the dots represents the number of DEGs associated with the GO term, and the colour of the dots represents the P-adjusted values

To investigate the molecular mechanism involved in modulating malaria parasitaemia, the key DEGs and signalling pathways between these two ICD subtypes were identified. DEGs analysis was conducted using the limma package in R, where the design matrix was constructed with model.matrix, linear modeling was performed with lmFit, empirical Bayes moderation was applied using eBayes, and differentially expressed genes were identified using topTable with the Benjamini–Hochberg adjustment method (adjust =‘BH’) and a p-value threshold of 0.05. Then 910 significant DEGs (294 upregulated and 616 downregulated) were identified in the subtype 1 group compared with the subtype 2 group with a filtering threshold of a p value less than 0.05 and |log2-fold change| greater than 0.5. The heatmap of the top 30 DEGs is shown in Fig. 3A. The volcano plot depicted in Fig. 3B shows annotations of the DEGs with a |log2-fold change| greater than 1.5. Then, GSEA was conducted on the 910 DEGs between the subtype 1 and subtype 2 groups using the clusterProfiler package. The hallmark GSEA result suggests that proliferation-related signalling pathways, including hallmark myc targets v1 and hallmark E2F targets, were activated, while inflammation-related pathways, including hallmark TNFA signalling via NFKB and hallmark inflammatory response, were suppressed in the subtype 1 group compared with the subtype 2 group (Fig. 4A). The KEGG GSEA results suggested that T-cell receptor signalling, antigen processing and presentation, natural killer cell-mediated cytotoxicity and cell adhesion molecules cams were activated in the subtype 1 group compared with the subtype 2 group (Fig. 4B). Moreover, GO enrichment analysis was applied to the upregulated and downregulated DEGs in the subtype 1 group compared with the subtype 2 group separately using the clusterProfiler package. The GO enrichment analysis results showed that the genes upregulated in the subtype 1 group were mainly involved in leukocyte cell‒cell adhesion, T-cell activation and differentiation and the T-cell receptor signalling pathway, while the downregulated genes were related to cytokine production, cytokine-mediated signalling pathways and regulation of the inflammatory response (Fig. 4C, D). These results suggest that the proliferative signalling pathway, T-cell receptor signalling pathway and T-cell activation and differentiation are upregulated in the subtype 1 group, while cytokine signalling and the inflammatory response tended to be upregulated In the subtype 2 group.

### Candidate ICD-associated hub genes that can differentiate between low and high parasitaemia were identified by protein–protein interaction (PPI) network construction and machine learning

**Fig. 5:**
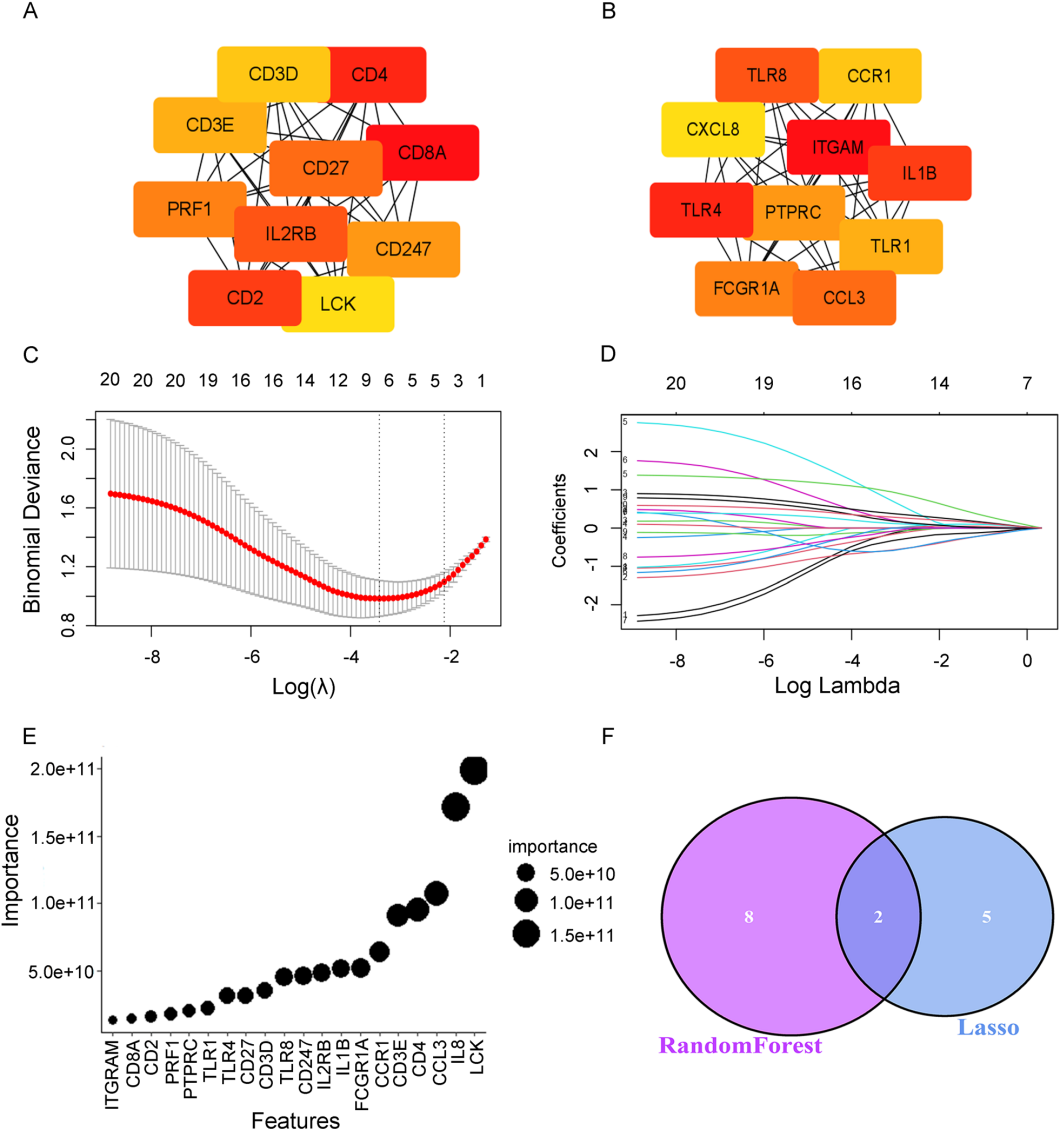
Candidate ICD-associated hub genes that can differentiate low and high parasitaemia were identified by protein‒protein interaction (PPI) network construction and machine learning. **A** The top 10 hub genes amongst genes upregulated in the ICD subtype 1 group were identified based on the maximal clique centrality (MCC) method using the cytoHubba plugin in Cytoscape. The color spectrum from red to yellow indicates the genes' connection degree, where darker hues signify a higher degree and greater gene importance. **B** The top 10 hub genes amongst genes downregulated in the ICD subtype 1 group were identified based on the MCC method using the cytoHubba plugin. **C** Binomial deviance was revealed by the LASSO regression model in the tenfold cross validation. The vertical dotted lines indicate the optimal values identified using the minimum and 1-SE criteria. **D** LASSO coefficient profiles of 20 selected ICD-associated genes in the tenfold cross-validation. **E** Twenty ICD-associated genes were ranked based on the importance of each gene associated with parasitaemia calculated by using RF machine learning algorithms. **F** Venn diagram showing the two candidate hub genes identified via both of the above algorithms

After confirming the existence of two ICD subtypes associated with parasitaemia in malaria infection, a PPI network was constructed to find hub genes for subsequent machine learning analyses. The PPI networks of the upregulated genes and downregulated genes in subtype 1 were established separately by using the STRING database. The results of the STRING analysis were imported into Cytoscape, and the top 10 hub genes for both upregulated genes and downregulated genes were identified based on the maximal clique centrality (MCC) method using the cytoHubba plugin (Fig. 5A, B). Furthermore, LASSO regression was applied to screen hub genes to differentiate low and high parasitaemia (Fig. 5C, D), and RF machine learning algorithms ranked the genes based on the calculation of the importance of each gene associated with parasitaemia (Fig. 5E). The intersection of the seven potential candidate genes from LASSO and the top 10 most important genes from the RF was visualized via a Venn diagram (Fig. 5F), and two genes (*CD3E* and *FCGR1A*) were retained for final validation.

### Development and validation of a nomogram for visualizing the correlation between ICD-associated hub genes and parasitaemia levels, and assessing its classification efficacy

**Fig. 6:**
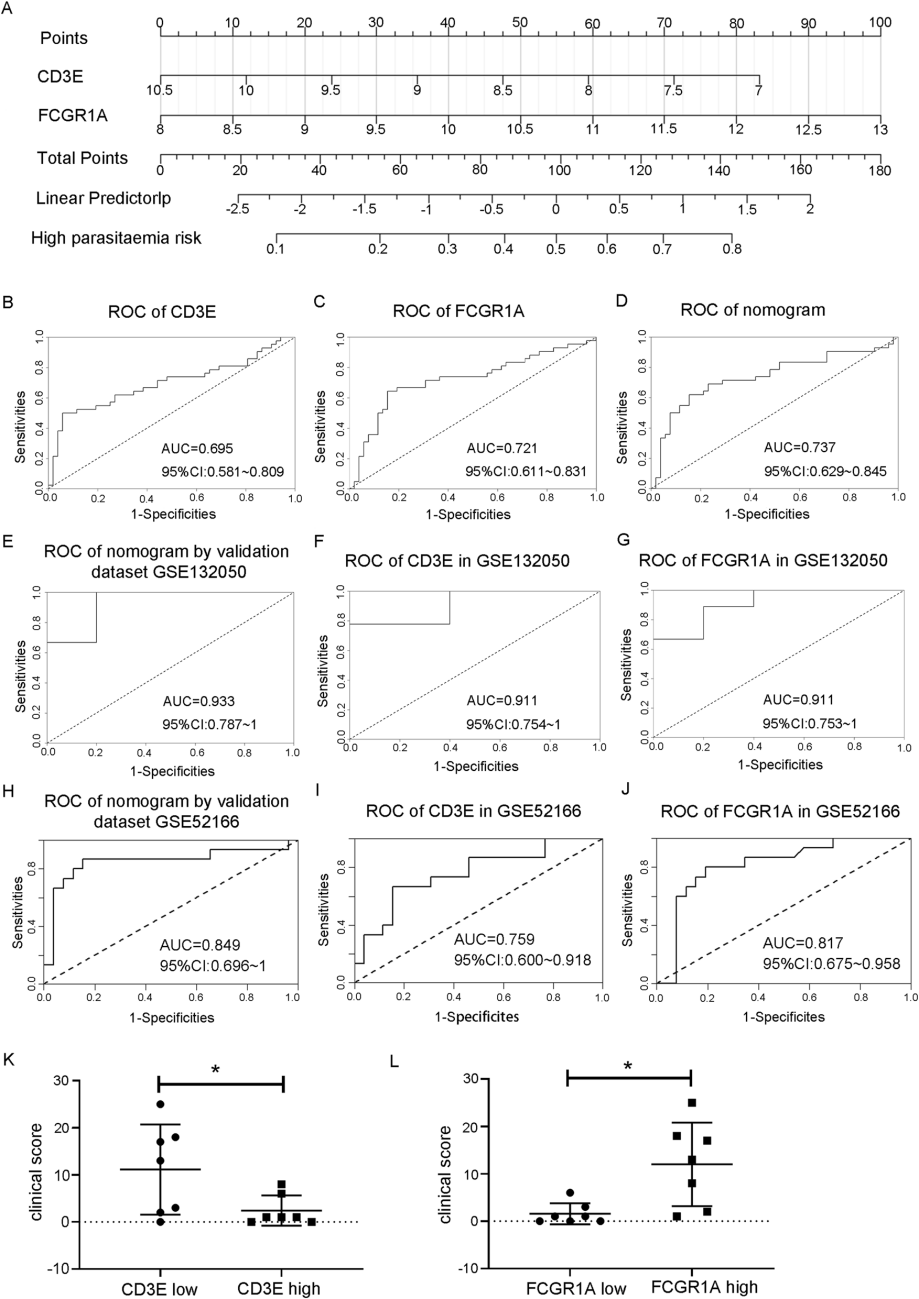
Nomogram construction and classification performance evaluation. **A** The nomogram for classification of high parasitaemia in the GSE34404 dataset. Each predictor variable (*CD3E* and *FCGR1A*) is represented on a separate axis. To use, assign points for each predictor value, sum these to get a total score, and then find the corresponding high parasitaemia risk on the bottom axis. **B**–**D** The ROC curves for each candidate hub gene (*CD3E* and *FCGR1A*) and the nomogram show their significant classification efficacy for high parasitaemia in the GSE34404 dataset. **E**–**G** The ROC curves of the nomogram and candidate hub genes (*CD3E* and *FCGR1A*) show their significant classification efficacy for high parasitaemia in the GSE132050 validation dataset. **H**–**J** The ROC curves of the nomogram and candidate hub genes (*CD3E* and *FCGR1A*) show their significant classification efficacy for high parasitaemia in the GSE52166 validation dataset. **K**–**L**The correlation between candidate hub gene (*CD3E* and *FCGR1A*) expression and clinical score (the sum of adverse events on the day of diagnosis) in the GSE132050 validation dataset. Statistical analyses were performed using the unpaired t test. * p < 0.05

A nomogram was constructed based on these two candidate hub genes (Fig. 6A), and a ROC curve was established to assess the classification specificity and sensitivity of each gene and the nomogram. As shown in Fig. 6B, C, the areas under the ROC curve (AUCs) for *CD3E* and *FCGR1A* were 0.695 and 0.721, respectively. The AUC value for the nomogram was 0.737, which showed the best classification performance (Fig. 6D). Then the nomogram's classification efficacy was validated in external cross-sectional studies GSE132050 and GSE52166, where it achieved AUC values of 0.933 and 0.849, respectively. This indicates robust classification performance across both training and validation cohorts (Fig. 6E–H). Additionally, in the external validation cohort GSE132050, both *CD3E* and *FCGR1A* hub genes exhibited identical AUC values of 0.911 (Fig. 6F–G). In cohort GSE52166, these genes demonstrated substantial classification utility with AUCs of 0.759 and 0.817, respectively (Fig. 6I–J). Furthermore, a significant inverse correlation was observed between *CD3E* expression and the clinical score, defined as the cumulative count of adverse events recorded on the day of diagnosis. Conversely, *FCGR1A* expression exhibited a significant positive correlation with the clinical score. These correlations were established based on data from the GSE132050 validation cohort (Fig. 6K–L). This result suggests that these two ICD-associated genes are correlated with the severity of malaria due to *P. falciparum* infection.

## Discussion

Immunogenic cell death (ICD) is a specific type of cell death that triggers an immune response by releasing danger signals and activating immune cells [18]. Currently, there is limited research on ICD in malaria. Thus, investigating ICD in malaria holds great promise for advancing the understanding of this disease, facilitating vaccine development and identifying new therapeutic targets.

In the present study, two ICD subtypes were identified by consensus clustering based on ICD-related gene expression in a cohort of patients infected with *Plasmodium falciparum*. Subtype 1 was found to be associated with lower parasitaemia and a better adaptive immune response than subtype 2. Functional enrichment analysis of DEGs between these two ICD subtypes showed that the proliferative signalling pathway, T-cell receptor signalling pathway and T-cell activation and differentiation were upregulated in subtype 1, while cytokine signalling and inflammatory response tended to be upregulated in subtype 2. In addition, two key DEGs between these two ICD subtypes were identified by PPI network and machine learning analyses, and a nomogram to classify high parasitaemia was constructed based on these two hub genes and evaluated by ROC curves. Finally, these two hub genes associated with ICD were found to be significantly correlated with the clinical manifestations (sum of the adverse events) of *P. falciparum* infection.

To date, the majority of research on ICD has been primarily focused on the field of oncology. 34 ICD-related genes that have been extensively summarized in the literature on tumour research were utilized to investigate the potential role of ICD in *Plasmodium* infection [12]. The result shows that, compared to controls without *Plasmodium* infection, infected patients exhibit upregulation of genes associated with cytokine production and downregulation of genes related to lymphocyte and monocyte differentiation. PCA conducted on the distribution of ICD genes demonstrated a significant disparity between malaria-infected and uninfected control samples. This significant alteration suggests a crucial role for immunogenic cell death in defence against malaria infection.

Two ICD subtypes were identified in the *P. falciparum* infection cohort. The subtype 1 group exhibited lower parasitaemia and a more robust adaptive immune response than the subtype 2 group. It was reported that when cells undergo ICD, they release DAMPs such as ATP, heat shock proteins, and DNA [19, 20]. These DAMPs can activate the immune system and facilitate the activation of antigen-presenting cells [21, 22]. Furthermore, ICD can also induce T-cell immune recognition and differentiation, enhancing immune effectiveness [2, 23]. The current study suggests that the subtype 1 group may be the ICD-activated group, which produces a more efficient immune response against malaria parasites. The subsequent functional enrichment analysis provided further evidence that the ICD-activated group exhibited upregulation of key pathways, including the T-cell receptor signalling pathway, T-cell activation and differentiation, and the proliferative signalling pathway.

In this study, two key DEGs, *FCGR1A* and *CD3E*, between the two subtypes of ICD in a *P. falciparum* infection cohort were identified using a PPI network and machine learning. The expression of these two DEGs was significantly correlated with malaria parasitaemia, and the expression of *FCGR1A* and *CD3E* was also significantly correlated with clinical manifestations. It has been reported that *FCGR1A* was upregulated at the protein level on both classical and non-classical monocytes during malaria [24]. And the activation of neutrophils, which also express *FCGR1A*, along with an increase in Neutrophil Extracellular Trap (NET) counts, has been observed to correlate with rising parasitaemia levels in *P. falciparum* infections. This phenomenon was particularly notable in cases of severe malaria [25]. However, the specific mechanisms of *FCGR1A* function in malaria infection are still relatively unknown. *CD3E* functions mainly as a part of the T-cell receptor, which, upon antigen binding, triggers the activation of T cells and the immune response. However, there is a lack of direct research on the specific role of CD3E in malaria infection. The identification of these hub genes may suggests a potential differential regulation of leukocyte subsets in the peripheral blood which may due to cell trafficking during malaria infection. The regulation characterized by an upregulation of T-cell activation and a simultaneous downregulation of neutrophils and monocytes may confer a more favourable environment for the clearance of *Plasmodium* parasites. The immune status of the host is a crucial factor that influences parasitaemia development after infection with malaria parasites. In this study, a nomogram was developed based on the expression levels of two ICD-related hub genes, revealing a significant correlation between the expression of two key host immune molecules, *FCGR1A* and *CD3E*, and *P. falciparum* parasitaemia. These two immune molecules may potentially serve as novel targets for the prevention and control of malaria, as well as offering new adjuvant targets for the development of malaria vaccines.

There were some limitations in this study. First, due to data limitations, the GSE34404 dataset, which contains the most cases that can be found, was chosen as the training dataset. The classification efficacy of the nomogram in the validation datasets GSE132050 and GSE52166 appeared notably high, potentially influenced by the limited sample sizes of these datasets. Further validation is recommended through an expansive, large-scale study encompassing a more substantial sample size. Second, although it was found that the ICD-associated genes *FCGR1A* and *CD3E* were associated with parasitaemia and clinical manifestations in *P. falciparum* infection, the biological or medical mechanisms underlying these phenomena remain unclear. Therefore, functional and mechanistic experiments are needed to verify and explain the roles of ICD in malaria infection.

## Conclusion

Our study delineated two distinct ICD subtypes in a *P. falciparum* infection cohort and identified two ICD-associated candidate hub genes, *FCGR1A* and *CD3E*, which are significantly correlated with parasitaemia levels and clinical manifestation following *P. falciparum* infection. This research can deepen the understanding of the human immune response induced by *P. falciparum* infection and provide adjuvant targets for malaria vaccine development.

### Supplementary Information

**Additional file 1: Figure S1.** GO enrichment of ICD genes with differential expression between the uninfected control group and malaria-infected group in the GSE34404 dataset. A. GO enrichment of ICD genes upregulated in the malaria-infected group compared with the uninfected control group. B. GO enrichment of ICD genes downregulated in the malaria-infected group compared with the uninfected control group.

**Additional file 2: Table S1.** The subsets of genes and immune cell types identified from the ImmPort database

## Data Availability

The datasets used and/or analysed during the current study are available from the corresponding author upon request.

## Abbreviations

ICD: Immunogenic cell death
DEGs: Differentially expressed genes
GEO: Gene expression omnibus
PCA: Principal component analysis
ssGSEA: Single-sample gene set enrichment analysis
NES: Normalized enrichment score
FC: Fold change
GO: Gene ontology
KEGG: Kyoto encyclopedia of genes and genomes
GSEA: Gene set enrichment analysis
STRING: The retrieval of interacting genes database
PPI: Protein–protein interaction
MCC: Maximal clique centrality
RF: Random forest
LASSO: Least absolute shrinkage and selection operator
ROC: Receiver operating characteristic curves
AUC: The area under the receiver operating characteristic curve values
CI: Confidence interval
DAMPs: Damage associated molecular patterns
BP: Biological process
CC: Cellular component
MF: Molecular function
NET: Neutrophil extracellular trap
PAC: Proportion of ambiguous clustering
