# Supplementary figures and images for: Classification and clinical significance of immunogenic cell death-related genes in Plasmodium falciparum infection determined by integrated bioinformatics analysis and machine learning
Source: Malar J. 2024 Feb 15;23:48. doi: 10.1186/s12936-024-04877-3 (PMC10868002; doi:10.1186/s12936-024-04877-3)

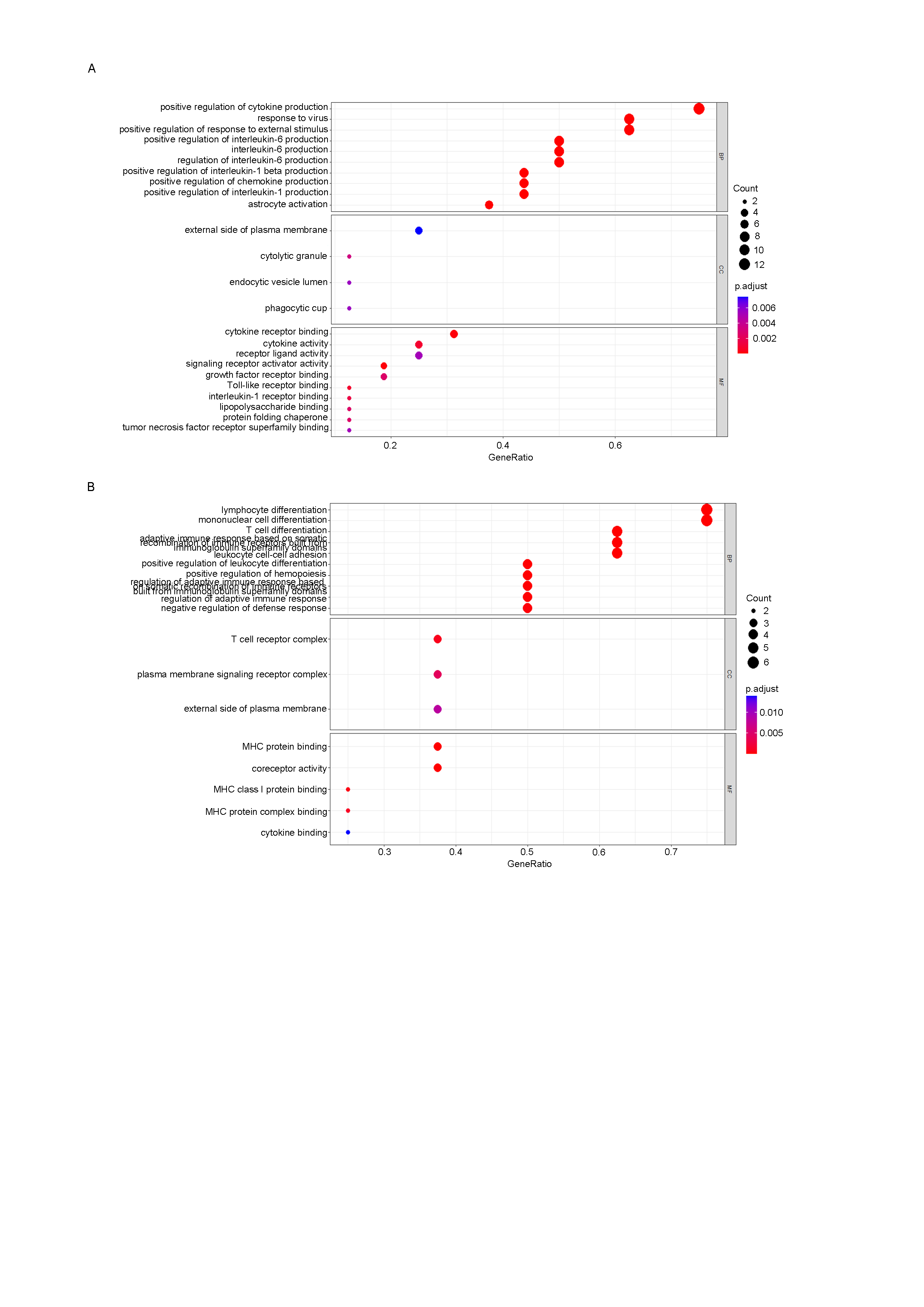

Supplement: Supplementary file 1 — Additional file 1: Figure S1. GO enrichment of ICD genes with differential expression between the uninfected control group and malaria-infected group in the GSE34404 dataset. A. GO enrichment of ICD genes upregulated in the malaria-infected group compared with the uninfected control group. B. GO enrichment of ICD genes downregulated in the malaria-infected group compared with the uninfected control group. [file 12936_2024_4877_MOESM1_ESM.tif]
